# Nutrition-sensitive agriculture programme impacts women’s mental health via food security in rural Bangladesh

**DOI:** 10.1136/bmjgh-2025-020509

**Published:** 2026-05-05

**Authors:** Thalia M Sparling, Cesar Cornejo, Claudia Offner, Axel Mayer, Jillian L Waid, Suneetha Kadiyala, Sabine Gabrysch

**Affiliations:** 1 London School of Hygiene & Tropical Medicine, London, UK; 2 Heidelberg Institute of Global Health, Heidelberg University, Heidelberg, Germany; 3 Department of Geography, University of Cambridge, Cambridge, UK; 4 Department of Psychological Methods and Evaluation, Bielefeld University, Bielefeld, Germany; 5 Member of the Leibniz Association, Potsdam Institute for Climate Impact Research (PIK), e. V., Potsdam, Germany; 6 Research, Learning, and Evaluation Unit, Helen Keller International, Dhaka, Bangladesh; 7 Institute of Public Health, Corporate Member of Freie Universität Berlin and Humboldt-Universität zu Berlin, Charité - Universitätsmedizin Berlin, Berlin, Germany

**Keywords:** Interdisciplinary Research, Nutrition, Mental Health & Psychiatry, Public Health, Global Health

## Abstract

**Introduction:**

Food insecurity and malnutrition, as well as poor mental health, negatively impact millions of people worldwide and can reinforce each other, compounded by gender inequity. Nutrition-sensitive agriculture interventions have the potential to improve these simultaneously. We analysed the impact of a homestead food production (HFP) programme on women’s mental health, including pathways through food insecurity, women’s empowerment and dietary diversity.

**Methods:**

The Food and Agricultural Approaches to Malnutrition cluster-randomised trial allocated 96 settlements in northeastern Bangladesh 1:1 to a HFP programme, implemented 2015–2018, and control. Data were collected at baseline in 2015, at endline in 2019/20 and continuously through a surveillance system. Depressive symptoms were assessed using the Edinburgh Postpartum Depression Scale (EPDS). We quantified the intervention’s impact on depressive symptoms at endline, analysing data from 2513 women using multilevel regression. We also examined whether that effect was mediated by household food security, women’s empowerment and women’s dietary diversity, using sequential mediation analysis with cluster-bootstrapped standard errors, adjusting for baseline covariates.

**Results:**

At baseline, 39% of households were severely food insecure and 69% of women did not have minimally diverse diets. At endline, 38% of women in the control and 32% in the intervention arm screened positive for depressive symptoms (EPDS≥12). The intervention reduced the odds of depressive symptoms by 23% (OR 0.77, p=0.03). There was no evidence that the combined pathway reduced depression (OR 0.95, p=0.24). When decomposed, food security was responsible for one-third of the total effect (OR 0.92, p=0.01). Most of the intervention effect on depression was through other pathways (OR 0.81, p=0.08).

**Conclusions:**

On average, intervention participants had better mental health 1 year after the programme ended, with some of the effect mediated by increased food security. There are likely other pathways through which nutrition-sensitive agriculture can improve mental health, such as social protection and income, which may act synergistically.

**Trial registration number:**

NCT02505711.

**WHAT IS ALREADY KNOWN ON THIS TOPIC**

- Mental health is associated with food security, diet quality and nutrition indices. However, it is less clear whether food and nutrition interventions can improve mental health, and even less is known about the mechanisms by which such improvements can occur.

**WHAT THIS STUDY ADDS**

- Our study shows that a homestead food production programme in rural Bangladesh improved depressive symptoms in women and that this was partly through improvements in food security.

**HOW THIS STUDY MIGHT AFFECT RESEARCH, PRACTICE OR POLICY**

- It should inspire further research into how food and nutrition activities can contribute to better mental health, and it strengthens the evidence base for integrated or multisectoral actions that can achieve wider health benefits.

## Introduction

 Food insecurity and undernutrition are leading causes of morbidity and mortality in low-income settings.^1^ Nutrition-sensitive agriculture programmes are designed to improve underlying determinants of food insecurity and undernutrition by addressing multiple components such as food production, nutrition education, women’s empowerment and income generation.^2 3^ Intended outcomes of such programmes are mainly improved household food security, maternal and child dietary quality and thereby child growth and development, with positive effects also along the impact pathway, including agricultural productivity, household income, women’s bargaining power, social networks and support, as well as climate resilience.^4^,^6^

Positive factors acting together can improve health and well-being overall,^7^ including mental health. Mental disorders have been the second leading cause of Years Lost to Disability in recent decades.^8^ Food security and nutrition have been empirically linked to mental health in various ways^9^,^11^: Poor mental health, especially common mental disorders such as depression and anxiety, can lead to reduced capacity for care (both of oneself and others), loss of motivation and lower productivity, which in turn can affect nutrition outcomes.^12^,^14^ In the reverse direction, food insecurity is known to cause stress, worry and depression.^15 16^ Poor diets and nutritional status (including specific micronutrients and diet quality as a proxy for micronutrient adequacy) have also been related to poor mental health, possibly through imbalances in neurotransmitters.^17^,^19^ Food security, nutritional status and mental health each share both determinants and outcomes, such as poverty, concomitant illnesses, gender and social disparity, violence and conflict.^20^,^23^

In South Asia and beyond, poor food security and nutrition as well as poor mental health are common and often cluster among groups and individuals with multiple vulnerabilities.^24^,^27^ In Bangladesh, the prevalence of screening positive for depressive symptoms among adults ranges from 6.5% to 31%, based on estimates from 32 studies.^28 29^ In 2024, an estimated 31% of Bangladeshi children aged 6–59 months were stunted, and about one quarter of the population were food insecure.^30^ Several studies have shown links between food insecurity, malnutrition and poor mental health in South Asia, largely focusing on the effects of maternal mental health on child (nutrition and health) outcomes,^31^,^34^ although the strength of the evidence varies between different populations and outcomes studied. Comparatively few studies have examined the effect of mental health on nutrition (or its proxies) in women. A study in Bangladesh (using baseline data from the same trial that this paper is based on) found that depressive symptoms in women (both perinatal and non-perinatal) were associated with food insecurity, poor diet quality and underweight.^35^

Even fewer studies have tried to pin down the causal effects of food security and nutrition on mental health, and almost none from lower-income settings have investigated the factors that mediate these relationships, including through women’s empowerment.^36^,^38^ Recently, though, some nutrition-sensitive agriculture interventions have shown improvements in women’s mental health, whether or not they included specific mental health components. In Tanzania, a randomised trial found that a participatory, peer-led agroecological intervention over 3 years (with components on sustainable farming, legume intensification, nutrition and women’s empowerment) improved mental health, at least in part via food security, but did not examine other factors in the mediation pathway.^39^ In Uganda, a trial of interpersonal therapy (a short-term, group-based psychotherapy for depression and interpersonal challenges) included nutrition-sensitive care groups in both treatment and control arms, which provided training on improved nutrition, hygiene and care practices for pregnant women, infants and young children. At first, the interpersonal therapy arm showed marked improvements in mental health. Over time, however, differences in mental health between arms equalised as both arms participated in the nutrition-sensitive care groups, possibly suggesting that these groups had an independent effect towards improving mental health.^40^

Given these emerging findings, food-related and nutrition-related interventions are worth investigating further for their contributions to improving mental health, especially as these interventions often focus on women. The multiple impact pathways within these complex interventions can thus be particularly important for women’s mental health. It is time to move towards causal evidence, and to understanding the pathways through which nutrition-sensitive agriculture programmes can improve mental health. This will help us understand the dynamics of multiple, intersecting health risks and disparities between individuals, households or communities, which could become intervention opportunities with synergistic benefits—especially important in light of slow progress towards several international health and development goals.^41^,^44^

With the aim to fill this research gap, we investigate the impact of a 3-year randomised nutrition-sensitive agriculture intervention on mental health in rural Bangladesh and quantify the degree to which observed effects were mediated by improvements in food security, women’s empowerment and dietary diversity.

## Methods

### Trial and setting

The Food and Agricultural Approaches to Malnutrition (FAARM) trial evaluated nutritional impacts of a homestead food production (HFP) programme on women and their children under 3 years (registered at https://clinicaltrials.gov/ct2/show/NCT02505711). The study site was in Nabiganj and Baniachong sub-districts of Habiganj district in Sylhet division, a rural, flood-prone area in north-eastern Bangladesh. The trial is described below, with further details in the study protocol.^45^

Communities, public leaders and local officials were involved at the inception of the trial, but neither participants nor the public were directly involved in the design, conduct, reporting or dissemination of our research.

The FAARM trial enrolled 2705 married women aged 30 years or younger in 96 settlements with access to at least 40 square metres of land for gardening. Most were enrolled at baseline, and 82 women who newly married into enrolled households were enrolled in the first year (online supplemental figure 1). The sample size was calculated for the primary trial outcome, length/height-for-age of children under 3 years of age at endline. Depression was not a pre-specified trial outcome and no further sample size calculations were undertaken for this analysis. After the baseline survey in 2015, the 96 settlements were allocated 1:1 to intervention (HFP programme) or control using covariate-constrained randomisation^46^ to ensure balance on key characteristics. Participants and data collectors were not informed of their allocation. Data analysts were not blinded to enable the project to track progress and adapt activities accordingly.

### Programme implementation

The intervention was implemented by the non-governmental organisation (NGO) Helen Keller International who has experience in implementing HFP programmes in Bangladesh for decades, aiming to improve year-round horticulture, small-animal husbandry and nutrition knowledge, and thereby improve nutrition outcomes.^47^,^49^ Their local implementation partner was the Voluntary Association for Rural Development, a national NGO.

The HFP programme implemented within the FAARM trial included four components: (a) nutrient-dense food production using improved agricultural practices in home gardens (eg, raised beds, natural pest control and organic fertilisers); (b) improved poultry production (eg, improved sheds and vaccination); (c) nutrition education (eg, trainings on infant and young child feeding (IYCF), sick child care and micronutrient-dense foods), as well as hygiene education; and (d) support for marketing surplus produce. The programme was delivered to groups of around 16 neighbouring women (range 8–26) formed at programme outset in 2015, with lead farmers designated in each group to support practices. Trainings comprised multiple group sessions annually, as well as ongoing individual counselling at home and small asset transfers (mainly seeds and subsidies for improved poultry shed construction) through 2018. At least 31 group sessions were offered, and attendance was generally high (~80% on average over 3 years).

### Data collection

All data were collected by teams of trained data collectors. The baseline survey was conducted in early 2015 and included anthropometry, blood sampling and interviews on household and socioeconomic characteristics, agricultural and animal husbandry, hygiene practices, women’s reproductive history, various empowerment aspects, as well as depressive symptoms, among others. The endline survey was conducted in late 2019 until early 2020, following a similar structure, including screening again for depression, covering certain women’s empowerment aspects and asking about food security.

In between baseline and endline, over 4 years, information on time-sensitive indicators was collected in both trial arms through the routine assessment component of a surveillance system. This included garden crop species diversity, poultry numbers and outputs, improved garden and poultry practices, household food security (twice), women’s empowerment (once towards the end of the trial) and dietary diversity (rotating, with each woman every 6 months, resulting in eight time points between baseline and endline). For pregnant and postpartum women and for young children, certain information was collected in addition or more often, such as IYCF indicators, dietary diversity and depression screening.

### Variables

The exposure of interest of this study was the random assignment to the intervention group versus control. The outcome of interest was screening positive for depression. This was measured with the Edinburgh Postpartum Depression Scale (EPDS), a 10-item psychometric scale (with a range of 0–30) used to detect probable clinical depression based on symptoms experienced in the last 7 days, with a focus on mood and not on somatic symptoms that may also be present during pregnancy.^50^ The EPDS is a commonly used tool that has been validated in many low-income contexts,^51^ including Bangladesh.^52^ Screening tools such as the EPDS are not designed to detect severity of symptoms, rather to indicate probability of being diagnosed with depressive disorder in a clinical assessment.^53^ The Bangladeshi validation of the EPDS established a cut-off of 10 for likely clinical minor or major depression combined.^52^ As there is no validated cut-off point for probability of major depression alone, we used ≥12 as a more conservative estimate, in line with other studies from South Asia.^35 54^ Our outcome was thus a dichotomous variable of a score of 12 or more in the EPDS, signifying probable depression.

We analysed three mediators: food security, a combination of women’s empowerment characteristics and dietary diversity, all of which may be independently related to mental health. We opted to average most available data on mediators to strengthen the power of the mediation analysis, even though this made the temporal sequence sometimes less straightforward. To operationalise food security, we calculated the average of three Household Food Insecurity Access Scale (HFIAS) categorised scores (range 1–4, with 1 indicating severe food insecurity and 4 indicating no signs of food insecurity)^55^ collected in late 2017, early 2019 and during FAARM endline in winter 2019/2020.

For women’s empowerment, we used nine variables partly adapted from Demographic and Health Surveys, measured in mid 2019 and at endline: social support score (ability to seek help and contact natal family), decision-making capacity score (ability to decide on various personal and household matters), husband communication score (ability to speak about personal and household matters with husband), external communication score (ability to speak to other women and publicly), network score (sum of relationships, topics discussed and support available from a random 5 women who live nearby), self-efficacy (perception of her capabilities and ability to reach her goals), mobility (whether a woman left the homestead in the past month), income (whether a woman earned any income in the past month) and income decision-making (whether a woman could decide on her own income). The last four are dichotomous indicators and the first five are scores which were rescaled to 0–2 (online supplemental table 1).

For dietary diversity, we used the standard Women’s Dietary Diversity Scale (WDDS) combined into 10 food groups from 21 food categories, counted only if women ate more than approximately 15 grams (~1 teaspoon) of a food.^56^,^58^ The WDDS ranges from 0 to 10, indicating the number of food groups eaten in the previous 24 hours.^56^ We detrended dietary diversity measures to minimise effects from Ramadan. To do this, we calculated the average effect of Ramadan on dietary diversity scores in a regression controlling for month, intervention arm and clustering, and then subtracted this from dietary diversity scores measured during Ramadan. We then calculated women’s dietary diversity as the average of five scores (from 2017 twice a year plus endline), corresponding to the time that the intervention was in full implementation.

Woman-level baseline covariates included EPDS score, dietary diversity score (WDDS), age (in years), highest educational level (from none to completion of high school or more), social support score, decision-making capacity score, husband communication score, external communication score, mobility and own income. Household-level baseline covariates included food insecurity category, asset score (in quintiles), religion (Muslim or Hindu), dependency ratio (the number of children (≤ 14) and elders (> 65) divided by number of household members between the ages of 15 and 65^59^), family type (joint or nuclear), and a count of garden crop species harvested in the previous year.

### Analysis

We used data from all women from whom depressive symptoms were measured at endline to examine the impact of being enrolled in an HFP intervention on depression in an intention-to-treat analysis. After describing women’s baseline characteristics by study arm, we quantified the intervention’s impact on screening positive for depressive symptoms using multilevel logistic regression models in Stata 16, without any covariates but accounting for clustering by settlement, and with those covariates that were also included in the mediation model (described below) but without imputed values, and the same model with imputed variables (table 2).

Our mediation hypothesis is based on three points: (a) that food security, dietary diversity and women’s empowerment can all contribute to mental health in distinct ways, even though there is some correlation between experiential constructs of food insecurity and low dietary diversity (eg, through sacrificing preferred foods or reducing intake of certain items) and (b) that dietary diversity does not cause women’s empowerment or food security. Rather, food security, as both a basic economic indicator of provision and an indicator of food consumption, precedes dietary diversity and is preserved at the expense of dietary diversity in line with Bennet’s Law.^60^ This is also supported by prevailing theoretical frameworks,^61^,^63^ as well as empirical literature unpacking cause and effect relationships between food security and dietary diversity.^64 65^ The third point (c) is that women’s empowerment can be a consequence of food security, in line with qualitative findings in our study area,^66^ and a driver of dietary diversity. Hence, we treated food security, women’s empowerment and dietary diversity as separate mediators, and ordered food security first, empowerment second and dietary diversity third in the sequential mediation pathway. We therefore investigated whether and to what degree the total observed effect of the intervention on depression symptoms was mediated by these pathways using a potential outcomes framework.^67^

We conducted multiple mediation analysis using sequential mediation en bloc^68 69^ with the medflex package in R.^70^ The key advantages of this approach for our analysis include (1) that it allows for causally-defined effects and effect decomposition in the context of binary outcomes and non-linear relationships, (2) that it does not require models for the mediators and therefore makes no restrictions regarding the (joint) distribution of mediators and covariates and (3) that it is not necessary to specify the interrelations and temporal ordering of the mediator components within a bloc. This model allowed us to calculate causally defined total, direct and indirect effects, as well as indirect effects for each mediator separately, under the assumption that there is no reverse causality in the mediator blocs.

The sequential approach requires the specification of three logistic models with the same set of covariates: Model A includes only the mediator food security (M1), Model B additionally includes the bloc of nine empowerment variables (M2) and Model C additionally includes dietary diversity (M3). The counterfactuals are then estimated using the imputation approach in medflex and the corresponding effects are obtained using natural effects models with covariates. We transformed total, direct and (specific) indirect effects on the log odds scale into ORs and reported those.

The total effect is computed based on Model C and is interpreted as the overall effect of the intervention via all pathways, that is simply the multiplicative change in odds of depression comparing the intervention and control group. The direct effect is also computed based on Model C and captures the effect of the intervention on the outcome that is not transmitted through any of the mediators. The indirect effect from Model C is an estimate of the effect via all mediators (ie, the effect on the outcome attributable to the difference between the mediator values in intervention vs control had all participants received the intervention).^71^ The M1-specific effect is the indirect effect obtained from Model A and represents the effect of the intervention through M1 (including pathways through M1 and subsequent mediators). The M2-specific effect is the contribution of the bloc of empowerment variables in addition to M1. It is obtained as the (exponentiated) difference between the indirect effects from Model B and A. The M3-specific effect is the contribution of M3 in addition to M1 and M2. It is obtained as the (exponentiated) difference between the indirect effects from Model C and B.

We used cluster-bootstrapped standard errors to account for the clustered design. Although the trial was well-balanced on most characteristics, we included baseline covariates to control for confounding on the mediator-outcome relationship, with the added benefit of increasing precision of estimates. In this sequential mediation, baseline covariates are included for the ‘bloc’, meaning that estimates of both mediation pathways are controlled for the set of confounders. Baseline covariates were chosen a priori from previous analyses and literature and retained regardless.

To account for missing data, we applied multiple imputation using chained equations (MICE) in R.^72^ At baseline, there were 95 missing EPDS, WDDS and HFIAS observations (3.7%), from women who were enrolled within the first year of the trial but after baseline. Missing observations were fewer for other variables (table 1). Most missingness resulted from absences during routine assessments of food security and dietary diversity (maximum missing per round was 14% and 18%, respectively). Although we have no reason to believe that data weren’t missing completely at random, and missingness was not associated with the outcome, we proceeded with imputation to increase the power of the mediation model. We used 30 iterations of 5 imputations, extracting the fourth imputation sequence (as it was best fit) to apply to the mediation model. We ran several sensitivity checks: the same analyses using only complete observations (without imputed data), and using different EPDS cut-offs.

**Table 1:** Baseline characteristics of women and households in the FAARM trial by intervention arm, for women with EPDS measured at endline (n=2513*)

Variable	Control (n=1255) mean or %	Intervention (n=1258) mean or %
Outcome characteristics at baseline		
Depressive symptom prevalence (EPDS≥12)	19.9%	20.1%
Mediating variable characteristics at baseline		
Household food insecurity category from HFIAS		
Severely food insecure	40.3%	36.7%
Moderately food insecure	9.0%	9.8%
Mildly food insecure	26.8%	28.0%
Food secure	23.8%	25.5%
Social support score (0=none to 2=most)	1.74	1.74
Decision-making capacity score (0=none to 2=most)	0.65	0.66
Husband communication score (0=none to 2=most)	1.78	1.79
External communication score (0=none to 2=most)	0.21	0.19
Mobility: Woman left home in previous month	31.8%	29.6%
Woman earned money in previous month	10.7%	10.5%
Women’s Dietary Diversity Score (WDDS, 1-10 food groups)	3.89	3.89
Woman characteristics		
Age in years	24.5	24.6
Educational category (1=none to 5=HSC+)		
None	16.8%	14.9%
Partial primary	21.2%	20.7%
Complete primary	23.5%	23.3%
Partial secondary	32.8%	34.7%
Complete secondary	3.9%	3.6%
HSC+	1.8%	2.9%
Household characteristics		
Wealth (household asset score quintile)		
Lowest	24.9%	21.1%
Low	23.6%	19.8%
Middle	19.6%	20.2%
High	16.1%	20.5%
Highest	15.7%	18.4%
Religion: Hindu (vs Muslim)	33.1%	29.1%
Family type: nuclear (vs joint)	33.6%	29.3%
Dependency ratio (ratio of children/elderly to working-age adults, range 0 to 4)	0.8	0.8
Number of garden crop species harvested (0–32)	5.8	6.5

*Up to 95 values missing from the baseline variables.

EPDS, Edinburgh Postpartum Depression Scale; FAARM, Food and Agricultural Approaches to Malnutrition; HFIAS, Household Food Insecurity Access Scale; HSC, high school certificate; WDDS, Women’s Dietary Diversity Score.

## Results

The participant flow is described in online supplemental figure 1. Of 2705 women enrolled, 2513 (93%) were reached at endline and answered the EPDS questions. At baseline, key characteristics were generally well balanced between arms, with small imbalances in household wealth (asset score in quintiles) and religion (Muslim or Hindu) (table 1). Food insecurity was common in this population, with 39% severely food insecure at baseline. Women’s dietary diversity was low, with less than four food groups (out of 10) consumed on average the previous day; and 69% not meeting minimum dietary diversity for women (Minimum Dietary Diversity of Women (MDD) ≥5 out of 10 food groups). The average age of participating women was just under 25 years at baseline, and 60% had received no secondary schooling. Women on average had good social support, but little decision-making capacity. For the mediators, we also present the averages from measures collected throughout the trial (as used in the mediation model), by intervention arm, with and without imputed values (online supplemental table 2).

At endline, 38% of women in the control and 32% in the intervention arm screened positive for depressive symptoms (EPDS≥12). Table 2 presents the intervention effects on depressive symptoms from multilevel logistic regression models, first accounting only for the clustered design, then including the same baseline covariates as in the mediation model, with and without imputed values. The intervention reduced the odds of depressive symptoms by roughly one quarter (OR 0.74, p=0.03). In addition, higher baseline depression score, higher dependency ratio and lower woman’s education were associated with depressive symptoms at endline (online supplemental table 3).

**Table 2:** Effect of a homestead food production intervention on women’s depressive symptoms at trial endline

Depression (EPDS≥12)	N	OR*	95% CI	P value
Model 1 (adjusted only for clustering by settlements)	2513	0.75	0.57 to 0.98	0.033
Model 2 (additionally adjusted for baseline covariates)†	2403	0.74	0.56 to 0.98	0.034
Model 3 (additionally adjusted for baseline covariates, with imputed values)†	2513	0.74	0.56 to 0.98	0.039

* ORs from multilevel logistic regression models with settlement-level random effects (96 settlements).

† Covariates included: baseline measure of woman’s EPDS score, household food insecurity score, dietary diversity score, age, educational category, social support score, decision-making capacity score, husband communication score, external communication score, mobility, own income, wealth, religion, dependency ratio, family type, garden crop species harvested.

EPDS, Edinburgh Postpartum Depression Scale.

**Figure 1:**
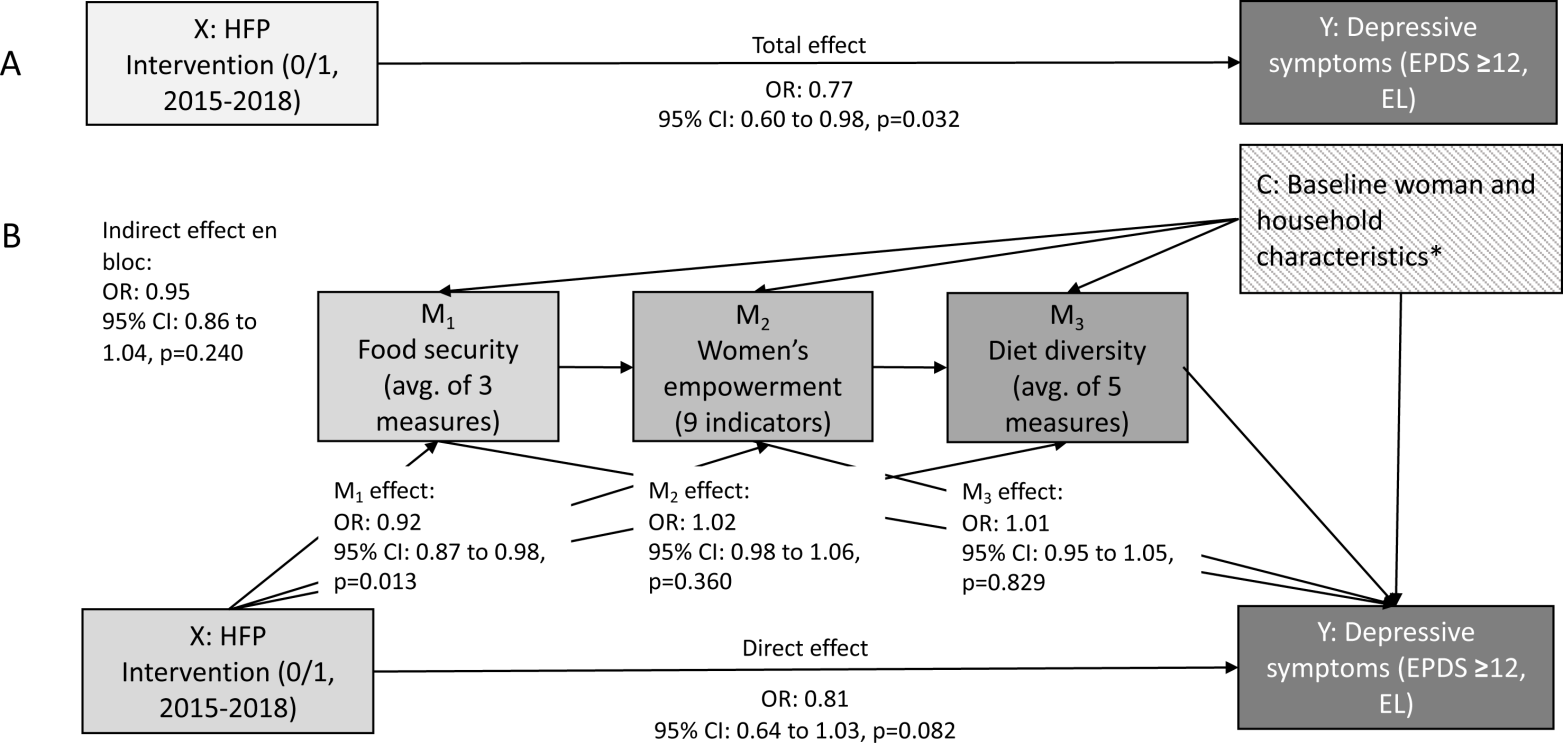
Diagram of the total effect (**A**) and direct and indirect effect (**B**) estimates of the impact of an HFP intervention in Bangladesh on women screening positive for depressive symptoms, mediated by household food security, women’s empowerment and women’s dietary diversity (n=2513). *Covariates included: baseline measure of woman’s EPDS score, household food insecurity score, dietary diversity score, age, educational category, social support score, decision-making capacity score, husband communication score, external communication score, mobility, own income, wealth, religion, dependency ratio, family type, garden crop species harvested. C, covariates; EL, endline; EPDS, Edinburgh Postpartum Depression Scale; HFP, homestead food production; M, mediator; X, exposure; Y, outcome.

In the mediation model (figure 1), the total intervention effect was very similar to the effect estimate in table 2, reducing odds of depression by 23% (OR 0.77), while including baseline covariates and using cluster-bootstrapped standard errors. There was no evidence of a mediating effect through all three sequential mediators (OR 0.95). By decomposing the mediators, we found that food security accounted for 35% (8/23) of the effect on depression (OR 0.92). Women’s empowerment and dietary diversity showed no independent mediating effect between the intervention and depressive symptoms at endline (OR 1.02 and 1.01, respectively). Most of the total effect of the intervention on depressive symptoms seemed to go through other pathways (OR 0.81). The total effect is the sum of the log odds from the indirect pathway en bloc and the direct effect (0.05+0.19 = 0.24, with the slight difference due to rounding from the log odds).

### Sensitivity analysis

For comparison, we ran the same mediation analysis as a complete case analysis with 2403 records and found very similar results and CIs to that with imputed values (online supplemental table 4). We also ran the same models using different cut-offs on the EPDS, given that there is no validated point that corresponds to screening positive for major depression in Bangladesh. Online supplemental table 5 shows depression prevalences in the FAARM study population at endline based on three different cut-off points with explanations. Effect estimates were similar between models, but compared with EPDS≥12, the evidence was somewhat stronger using EPDS≥11 and weaker using EPDS≥13.

## Discussion

We found that a 3-year HFP programme as part of the FAARM cluster-randomised trial reduced depressive symptoms in Bangladeshi women 1 year after the programme had ended. Food security, but not women’s empowerment or dietary diversity, accounted for about one-third of the observed effect on mental health, while most of the effect of the HFP intervention on depression was realised through other factors. Although the intervention included various nutrition-sensitive activities, it did not include components directly aimed at mental health.

To our knowledge, this is the first analysis to examine the impact of HFP on mental health; it is also the first analysis to examine the impact of a nutrition-sensitive agriculture intervention on mental health in South Asia, and the first such pathway analysis. Our findings are similar to the only other analysis of this kind, which found that an agroecological intervention in Tanzania (Singida Nutrition and Agroecology Project, SNAP-Tz) reduced depression (OR 0.57), and that about a quarter of the observed effect was due to improvements in food security, leaving 77% of the effect due to other pathways.^39^ They found that domestic violence, lack of task sharing in women’s work and decision-making on income allocation (in the subgroup with higher scores) were all related to depressive symptoms, but did not examine these or dietary diversity in their mediation analysis.

In addition to food security, we investigated both dietary diversity and women’s empowerment as potential pathways through which the HFP intervention may have reduced depression, but neither seemed to play any role. The intervention improved women’s dietary diversity^58^ and more diverse diets were linked to less depression in our setting at baseline,^35^ making it a good candidate. Yet for mediating the intervention effect on depression, it seems that dietary quality in the individual was of less importance than the ability of the household as a whole to obtain a standard diet. The WDDS indicator is highly standardised and was measured multiple times over the trial, making measurement error less of a concern.

Although indicators for women’s empowerment are less well established,^71^ we explored it as an additional mediator given that women’s empowerment is a possible candidate on theoretical grounds. While we had found earlier that the HFP intervention improved women’s agency in the subset evaluated in the project-level Women’s Empowerment in Agriculture Index (pro-WEAI) sub-study,^73^ there was little impact on any of these indicators that we measured close to or at endline, when measured in the full trial population – which can explain the lack of effect through women’s empowerment in this study. It is possible that a different timing or different indicators would have yielded a different result, and our findings thus do not exclude the possibility of a mediating effect of empowerment.

Our analysis adds to emerging findings that nutrition-sensitive interventions can improve other important health outcomes besides nutrition. Furthermore, this analysis contributes to the growing—although mostly observational—literature showing the broader relationship of food security and nutrition with mental health.^74^ For instance, it is established that food insecurity is almost always associated with worse mental health outcomes, but evidence for causality has been lacking, and mechanisms through which these are linked (eg, poverty, worry and stress, stigma, violence against women, diets and nutrition) have hardly been explored.^15^

It will be useful to investigate whether food security, women’s empowerment and diet quality impact women’s mental health in other settings. Some indicators of women’s agency have been linked to other outcomes, such as dietary diversity, in this study site, while others were not related.^73 75^ The group structure of the HFP training, increased resources controlled by women or jointly owned in the household, women’s improved knowledge and increased interaction within women’s groups and communities are likely important components that can improve both women’s mental health and nutrition.^73 76^ If the intervention had led to marked improvements in social support, decision-making, household dynamics and spousal relationships, these might have played a greater role as mediators. As a substantial part of the impact could not be explained in our study, it will also be important to examine further hypotheses about the impact pathways from HFP to women’s mental health in future analyses. Other stressors such as stigma, family income and financial stability would also be worthy of investigation in similar analyses as mediating and moderating factors. Establishing causality and unpacking specific pathways could help design even more effective programmes in terms of their impact on mental health.

There is increasing recognition that improving mental health requires approaches beyond conventional cognitive and behavioural therapies.^43 77^ Our findings contribute to this perspective by demonstrating that HFP—an intervention not traditionally designed as a mental health programme, but to reduce malnutrition—can improve women’s mental well-being. Conversely, mental health has been proposed as a determinant of food security and nutrition outcomes,^3^ which is supported by empirical research.^78^,^80^ Better mental health, especially for women, can have even wider health benefits in lower-income settings, including, for example, dietary choices and early childhood development.^10 16 34 81 82^ This implies that interventions like HFP may generate benefits well beyond their primary objectives. These should be measured and accounted for when evaluating the most cost-effective public health strategies.

Taken together, there is evidence that supports relationships in both directions between food security/nutrition and mental health, and it is reasonable to conclude that they are interlinked and synergistic.^1783^,^88^ Much of the literature on these relationships focuses on perinatal women given their heightened risk and implications of women’s mental health for children,^74 89 90^ although there have also been studies in general female populations.^15 91^ Globally, women are about 1.5 times more likely than men to experience depression^92^—and depression prevalence in the perinatal period is even higher than in the general female population.^93^ Furthermore, women often have a prominent role in family’s food procurement and preparation and caretaking and are more likely to experience food insecurity.^94^ Based on this, our findings suggest that policies and programmes should strive to integrate food security, nutrition and mental health actions that could lead to increased effectiveness and co-benefits for each. With almost one in three people food insecure, and approximately one in 11 suffering from chronic hunger,^95^ while an estimated 13% of the global population experience mental health problems,^96^ identifying intervention components that work together to address these elusive and widespread health issues simultaneously would be a key step towards integrated well-being interventions.

While this was not part of our objectives to study, we found a notable increase in the prevalence of depression among women from baseline (control and intervention 20%) to endline (control 38%; intervention 32%). There are several possible reasons. It is conceivable that the true prevalence did increase across both groups, and the increase was mitigated by the HFP intervention. Another possibility is that there was measurement bias; the data collection team at baseline may have performed differently than the surveillance team that collected the depression data until endline, although both received training and course correction. The surveillance data collectors worked in the study area for much longer, making repeated visits to the women, who may have become more familiar both with the interviewers and/or the EPDS questions. This familiarity could have changed women’s responses, most likely in the direction of being more willing to admit to problems, which could explain the higher depression prevalence at endline compared with baseline.

This study benefits from a well-balanced, randomised intervention design and rigorous data collection standards. While depression was not a pre-specified trial outcome, with 2705 enrolled women in 96 clusters, the study is well-powered to detect differences in depressive symptoms, which are highly prevalent in this setting. We measured depressive symptoms around 1 year after intervention activities ended, which enabled us to examine persistent benefits from HFP on mental health. We managed to reach 93% of participants at endline, more than 4 years after enrolment, thus limiting the scope for selection bias. The continued and detailed data collection between baseline and endline in both study arms allowed us to examine the role of mediators. Having multiple measures of household food insecurity and women’s dietary diversity over time reduces measurement error, as the aggregate measures for each woman are more precise, and differences between data collectors are evened out. Having detailed baseline measures allowed us to control for mediator-outcome confounding.

There are several limitations of this study. We used a simple screening tool as a proxy for depression, which can suffer from rater and response bias, especially when collected by different people or teams. We were unable to carry out further clinical assessment, or multiple measures over time in the full sample, which would have strengthened the findings. There was no validated cut-off point in our setting, but effect estimates were consistent across sensitivity checks. Unlike for food security and dietary diversity, women’s empowerment was only measured once toward the end of the trial. Non-differential measurement error in mediators would reduce their mediation effect. Another limitation is the lack of blinding of participants which can lead to bias. While data collectors were blinded, it is possible that they observed intervention activities and were thus unblinded to allocation, potentially leading to bias. Our sample size is relatively large; nevertheless, a higher precision of our estimates would have been desirable. In the mediation analysis, we assumed that there was no reverse causality in the mediators and that the order of mediators was correct. Finally, we did not assess or disentangle other potential pathways through which the intervention may have impacted depressive symptoms.

## Conclusion

We found that women participating in a 3-year HFP intervention as part of the FAARM trial had better mental health than controls 1 year after the intervention ended, due in part to increased food security. We conclude that nutrition-sensitive interventions, even without direct mental health components, can play a role in improving mental well-being. The evidence base could be further strengthened by examining these relationships in other settings and programmes. The most conclusive research would come from controlled, experimental or quasi-experimental data on programmes or policies which measure pathway indicators through repeated monitoring or surveillance systems. There are likely other pathways through which nutrition-sensitive agriculture activities can improve depression and mental health generally, such as increased social and financial resources, social protection and income, which may even act synergistically towards improvements in wider health outcomes. Further research should explore these and additional pathways that lead to improvements in health.

Our findings offer practical implications: participating in a nutrition-sensitive agriculture intervention improved mental health without intending to. Thus, by strengthening programme components that also improve mental health (eg, components that contribute to food security), and paying attention to synergies between programme components, we may be able to achieve stronger improvements in mental health and even other health outcomes. Overall, this study helps us understand part of the mechanisms by which nutrition-sensitive programmes can improve mental health, highlights important areas for research to elucidate these relationships further, and offers some guidance on how programmes could be strengthened to achieve more and wider health impact.

## Supplementary material

10.1136/bmjgh-2025-020509 online supplemental file 1

## Data Availability

Data are available upon reasonable request.
