# Supplementary material for: Nutrition-sensitive agriculture programme impacts women’s mental health via food security in rural Bangladesh
Source: BMJ Glob Health. 2026 May 5;11(5):e020509. doi: 10.1136/bmjgh-2025-020509 (PMC13150902; doi:10.1136/bmjgh-2025-020509)
Supplement: online supplemental file 1 [file bmjgh-11-5-s001.pdf]

Manuscript title: *Nutrition-sensitive agriculture programme impacts women's mental health via food security in rural Bangladesh*

Supplementary files

Supplementary Figure 1*: Study flowchart of clusters and participants with timing of enrolment and assessments*

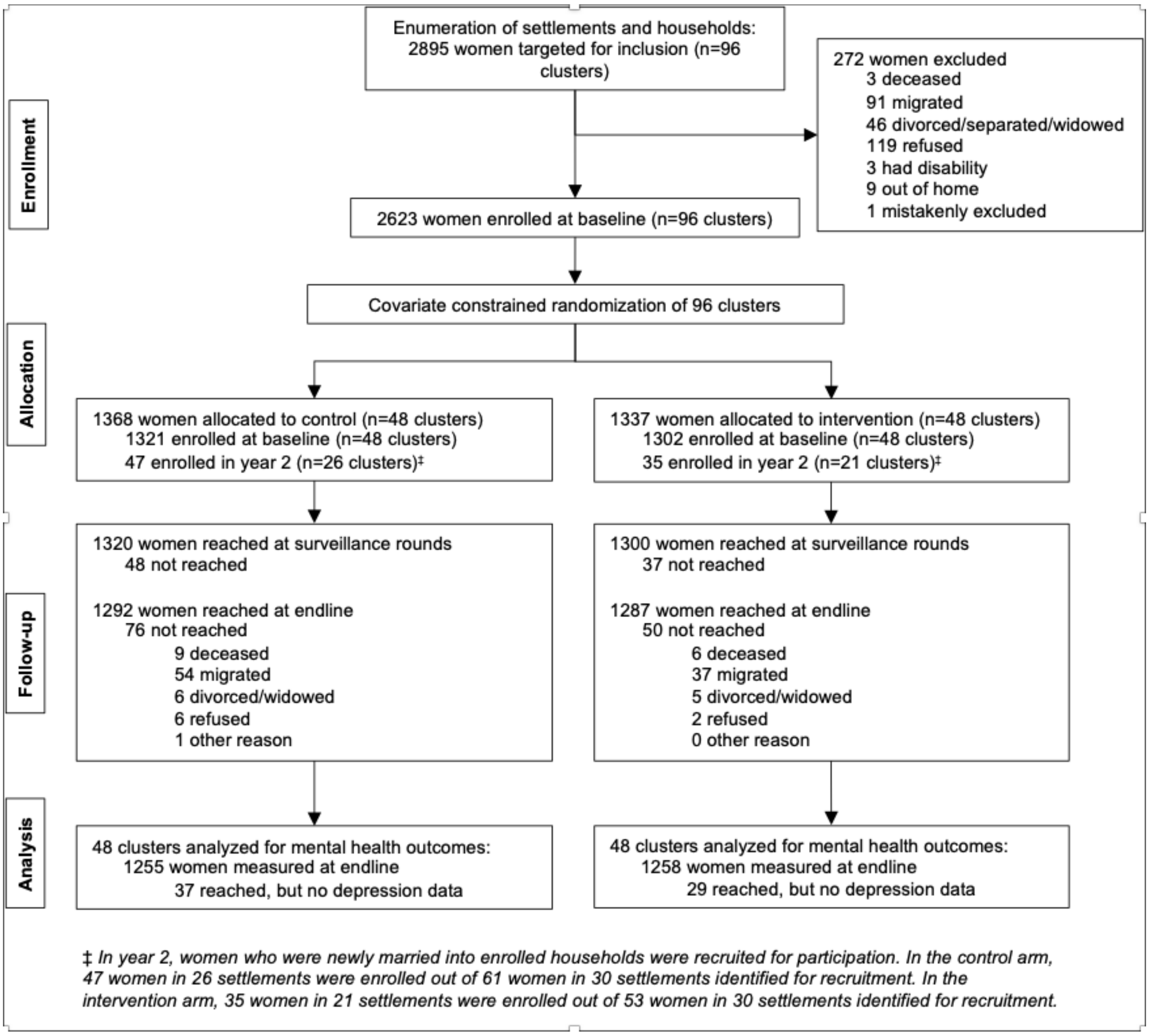

Supplementary Table 1: Construction of women's empowerment variables

Empowerment domain		Score*
**Social support**		
Has a source for help:<br>• With a cash loan<br>• With a food loan<br>• If sick<br>• Talking about problems	1 point for each positive answer	0-2, rescaled from 0-5
Has frequent contact with natal family	1 point for often (vs. sometimes or never)	
**Decision-making**		
Ability to decide on issues:<br>• Food preparation<br>• Large purchases<br>• Daily purchases<br>• Visiting her natal family<br>• Health matters	1 point for each issue if woman is listed as a decision-maker (can be jointly with others)	0-2, rescaled from 0-5
**Communication with husband**		
Topics regularly discussed with husband:<br>• Work/agricultural activity<br>• Events at home<br>• Expenditures<br>• Community events<br>• Woman's own health	1 point for each topic discussed often or sometimes (vs. never)	0-2, rescaled from 0-5
**External communication**		
Comfort level speaking out on issues	1 point for fairly comfortable or very comfortable (vs. rather uncomfortable or uncomfortable)	0-2, rescaled from 0-6
Usually discusses the following topics with other women:<br>• Problems of the community<br>• Education problems<br>• Health problems<br>• Women's issues<br>• Information on health or nutrition	1 point for each topic	
Social network score		
• Number of women known<br>• Number of women spoken with<br>• Number of women who could give support<br>• Number of women with whom they discuss:<br>○ gossip<br>○ health or nutrition<br>○ family matters<br>○ business<br>○ agriculture<br>○ religion<br>○ education<br>○ social issues	Sum of the number of relationships women had, topics discussed, and support available from a random 5 women who live nearby the women surveyed	0-2, rescaled from 0-53

◦ gardening or poultry rearing<br>• Number of women they greet formally<br>• Number of women they could ask for a loan<br>• Number of women they can ask for help when sick<br>• Number of women with whom they can talk about their problems		
**Self-efficacy****		
A woman's perception of her capabilities and ability to reach her goals based on the sum score of 8 statements***, each scored 1-5: strongly disagree (1), disagree (2), neither agree or disagree (3), agree (4), or strongly agree (5). Score of 0-40.	1 point if score is 32 or higher	0/1
**Mobility**		
Woman left the homestead in the previous month	1 point for positive answer	0/1
**Own income****		
Woman earned any money in the previous month	1 point if more than 0 Taka	0/1
**Income decision-making**		
Woman decides on her own earnings**	1 point if woman is listed as a decision-maker (0 if woman is not a decision-maker or has no earnings)	0/1

** Scale variables were rescaled by dividing through the maximum number of possible points and then multiplying by 2. **Available only at endline.*

***Statements: 1) I will be able to achieve most of the goals that I have set for myself, 2) When facing difficult tasks, I am certain that I will accomplish them, 3) In general, I think that I can obtain outcomes that are important to me, 4) I believe I can succeed at most any endeavor to which I set my mind, 5) I will be able to successfully overcome many challenges, 6) I am confident that I can perform effectively on many different tasks, 7) Compared to other people, I can do most tasks very well, 8) Even when things are tough, I can perform quite well. Reference: Chen G, Gully SM, Eden D. Validation of a new general self-efficacy scale. Organizational research methods. 2001 Jan;4(1):62-83.

Manuscript title: *Nutrition-sensitive agriculture programme impacts women's mental health via food security in rural Bangladesh*

Supplementary Table 2: Mediator values over the FAARM trial by intervention arm (with and without imputed values) for women with EPDS measured at endline (n=2513)

Variable	Control (n=1255) mean or %	Intervention (n=1258) mean or %
**a) Without imputed values**		
**Household Food Insecurity category** (average of late 2017, early 2019 and endline**)**		
Severe food insecure	11.6%	8.5%
Moderate food insecure	27.6%	23.3%
Mild food insecure	35.1%	34.5%
Food secure	25.6%	33.8%
**Dietary diversity score** (4 surveillance rounds 2017-2019 and endline, de-trended for Ramadan)		
Average dietary diversity score	4.44 ± 0.86	4.89 ± 0.99
**Empowerment** (one measure in mid 2019 or endline)		
Decision-making capacity score (0-2)	1.16 ± 0.66	1.12 ± 0.67
External communication score (0-2)	1.62 ± 0.42	1.67 ± 0.41
Communication with husband score (0-2)	1.94 ± 0.22	1.93 ± 0.24
Social support score (0-2)	1.85 ± 0.36	1.85 ± 0.36
Social network score (0-2)	0.30 ± 0.29	0.41 ± 0.35
Self-efficacy (score ≥32)	88.3%	89.2%
Mobility: Woman left homestead in previous month	42.0%	45.0%
Woman earned money in previous month	13.7%	18.0%
Woman can decide on her own income	24.5%	31.4%
**b) With imputed values**		
**Household Food Insecurity category** (average of late 2017, early 2019, and endline)		
Severe food insecure	10.1%	7.7%
Moderate food insecure	28.1%	23.3%
Mild food insecure	35.7%	34.8%
Food secure	26.1%	34.2%
**Dietary diversity score** (4 surveillance rounds 2017-2019 and endline, de-trended for Ramadan)		
Average dietary diversity score	4.45 ± 0.81	4.90 ± 0.94
**Empowerment** (one measure in mid 2019 or endline)		
Decision-making capacity score (0-2)	1.16 ± 0.66	1.13 ± 0.67
External communication score (0-2)	1.63 ± 0.42	1.67 ± 0.41
Communication with husband score (0-2)	1.94 ± 0.21	1.93 ± 0.23
Social support score (0-2)	1.86 ± 0.35	1.85 ± 0.35
Social network score (0-2)	0.30 ± 0.29	0.41 ± 0.35
Self-efficacy (score ≥32)	88.3%	89.2%

Mobility: Woman left homestead in previous month	42.3%	44.9%
Woman earned money in previous month	13.2%	17.2%
Woman can decide on her own income	24.5%	31.4%
Values are % for categorical or binary variables or means ± standard deviations for continuous variables.<br>*FAARM: Food and Agricultural Approaches to Reducing Malnutrition;*<br>*EPDS: Edinburgh Postpartum Depression Scale.*		

Manuscript title: *Nutrition-sensitive agriculture programme impacts women's mental health via food security in rural Bangladesh*

Supplementary Table 3: Effects of the intervention and of baseline covariates on depression among women enrolled in the FAARM trial in rural Bangladesh*

**Variable**	**OR**	**95% CI**	**p-value**
HFP intervention	0.74	0.56 to 0.98	0.039
Baseline depressive symptom score	1.07	1.05 to 1.09	<0.001
Woman's age	0.99	0.97 to 1.02	0.615
Religion (Muslim vs. Hindu)	0.79	0.61 to 1.02	0.073
Family type (joint vs. nuclear)	1.08	0.85 to 1.36	0.536
Dependency ratio	1.27	1.06 to 1.53	0.011
Wealth quintile			
Lowest	Reference		
Low	1.01	0.77 to 1.33	0.937
Middle	1.10	0.81 to 1.48	0.538
High	1.13	0.82 to 1.58	0.452
Highest	1.05	0.73 to 1.52	0.804
Women's educational category			
None	Reference		
Partial primary	0.96	0.71 to 1.28	0.758
Complete primary	0.89	0.66 to 1.19	0.427
Partial secondary	0.72	0.54 to 0.97	0.029
Complete secondary	0.55	0.31 to 0.97	0.040
High School Certificate or higher	0.66	0.34 to 1.29	0.228
Number of crops harvested	1.00	0.98 to 1.03	0.650
Household Food Insecurity Access Scale			
Severely food insecure	Reference		
Moderately food insecure	1.01	0.73 to 1.39	0.962
Mildly food insecure	1.06	0.83 to 1.34	0.653
Food secure	0.92	0.70 to 1.20	0.537
Women's Dietary Diversity Score	1.02	0.95 to 1.09	0.592
Social support score	1.01	0.81 to 1.27	0.933
Husband communication score	1.00	0.79 to 1.28	0.981
External communication score	1.23	0.96 to 1.59	0.100
Decision-making capacity score	0.87	0.73 to 1.04	0.117
Woman left homestead in previous month	1.10	0.90 to 1.34	0.345
Woman earned no money in previous month	0.92	0.69 to 1.22	0.544

**Estimates are from a multivariable multilevel logistic regression model with random effects at the settlement level and depression defined as EPDS ≥12 (n=2513)*

FAARM = Food and Agricultural Approaches to Reducing Malnutrition, HFP = Homestead Food Production, EPDS = Edinburgh Postpartum Depression Scale; OR = Odds ratio; CI = Confidence interval

Manuscript title: *Nutrition-sensitive agriculture programme impacts women's mental health via food security in rural Bangladesh*

Supplementary Table 4: Effect estimates for intervention impact on screening positive for depression, mediated by household food security and dietary diversity, without imputed values, n=2403

**Effect**	**Odds Ratio**	**Log odds**	**95% Confidence Interval**	**p-value**
Total	0.77	-0.26	0.60 to 0.99	0.04
Natural direct	0.82	-0.20	0.64 to 1.04	0.10
Natural indirect	0.95	-0.05	0.86 to 0.99	0.27
M1: Household food insecurity category	0.92	-0.08	0.87 to 0.98	0.01
M2: Women's empowerment	1.01	0.01	0.96 to 1.07	0.56
M3: Women's dietary diversity score	1.01	0.01	0.96 to 1.06	0.70

Manuscript title: *Nutrition-sensitive agriculture programme impacts women's mental health via food security in rural Bangladesh*

Supplementary Table 5: Prevalence for likely major depression based on three different cut-off points on the Edinburgh Postpartum Depression Scale among FAARM trial participants in rural Bangladesh at endline, with rationale for each

**Cut-off on EPDS**	**Rationale**	**Prevalence of screening positive (n=2513)**
≥11	Validated cut-off for probability of minor and major depression in Bangladesh	39%
≥12	Often used in South Asia and other LMIC contexts as the best cut-off for probability of major depression	35%
≥13	Used on the original validation study (Cox et al., 1996 (51)), but not validated in Bangladesh	30%
FAARM: Food and Agricultural Approaches to Reducing Malnutrition<br>*EPDS: Edinburgh Postpartum Depression Scale*		
